# Supplementary material for: The interactome of intact mitochondria by cross-linking mass spectrometry provides evidence for coexisting respiratory supercomplexes
Source: Mol Cell Proteomics. 2017 Dec 8;17(2):216–32. doi: 10.1074/mcp.RA117.000470 (PMC5795388; doi:10.1074/mcp.RA117.000470)
Supplement: Supplemental Data [file supp_RA117.000470_133922_0_supp_23978_fzffwf.zip › spectra_annotation/mito_spectra_annotation/359-1-3-1-11-1.pdf]

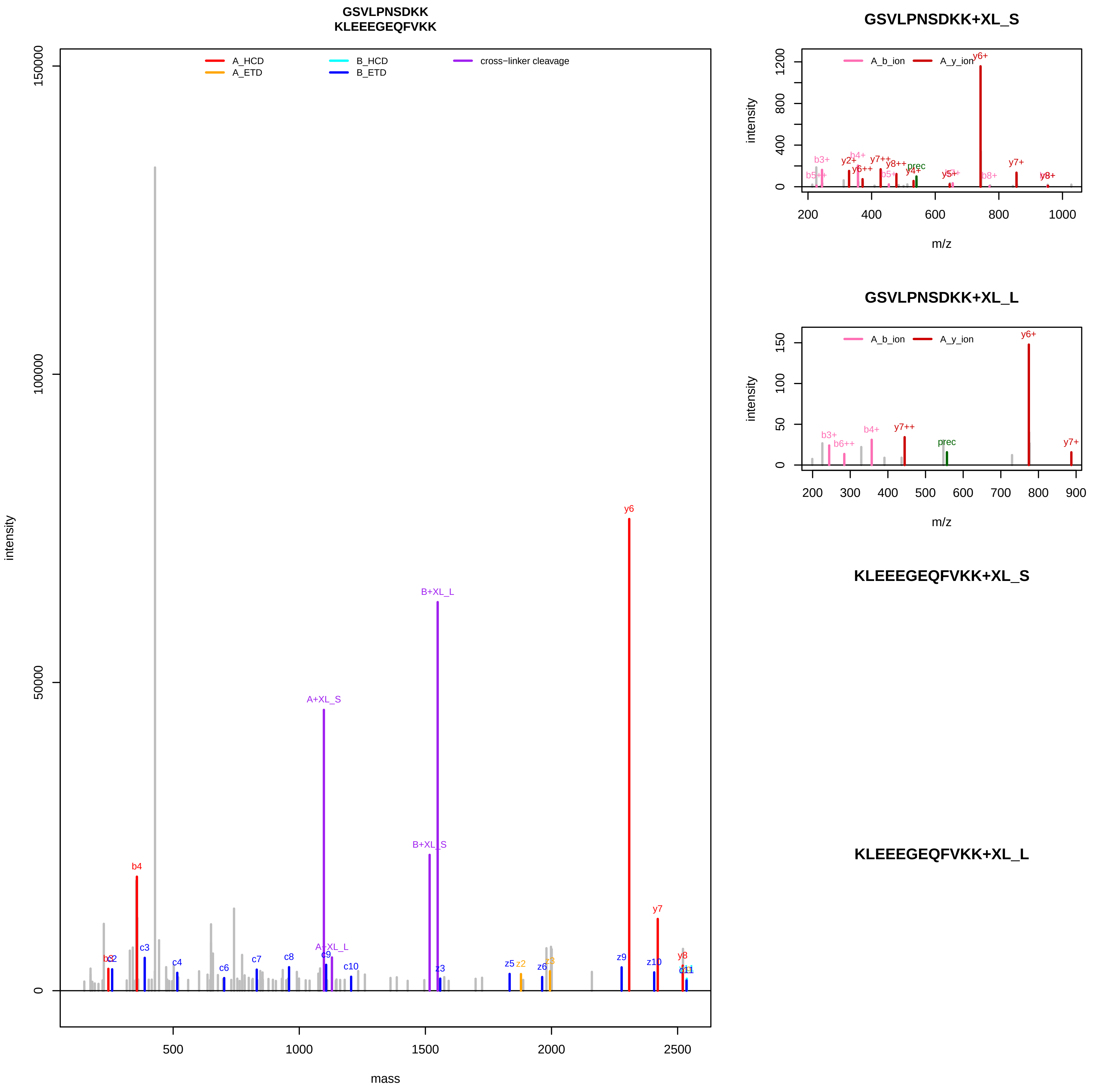
GSVLPNSDKK
KLEEEGEQFVKK
A_HCD
A_ETD
B_HCD
B_ETD
cross−linker cleavage
intensity
mass
0
50000
100000
150000
500
1000
1500
2000
2500
b4
c3
c4
c6
c7
c8
c9
c10
A+XL_S
A+XL_L
B+XL_L
B+XL_S
z3
z5
z2
z6
z9
z10
y6
y7
y8
c11
GSVLPNSDKK+XL_S
A_b_ion
A_y_ion
intensity
m/z
0
400
800
1200
200
400
600
800
1000
b3+
b5++
y2+
b4+
y6++
y7++
y8++
prec
y4+
b5+
y5+
y6+
b8+
y7+
y8+
GSVLPNSDKK+XL_L
A_b_ion
A_y_ion
intensity
m/z
0
50
100
150
200
300
400
500
600
700
800
900
b3+
b6++
b4+
y7++
prec
y6+
y7+
KLEEEGEQFVKK+XL_S
KLEEEGEQFVKK+XL_L
